# Foetal blood flow measured using phase contrast cardiovascular magnetic resonance – preliminary data comparing 1.5 T with 3.0 T

**DOI:** 10.1186/s12968-015-0132-2

**Published:** 2015-04-18

**Authors:** Beverly Tsai-Goodman, Meng Yuan Zhu, Mashael Al-Rujaib, Mike Seed, Christopher K Macgowan

**Affiliations:** Department of Paediatric Cardiology, Bristol Children’s Hospital, University of Bristol, Bristol, United Kingdom; Department of Paediatrics and Division of Cardiology, University of Toronto & Hospital for Sick Children, Toronto, Canada; Institute of Medical Science, University of Toronto, Toronto, Canada; Department of Medical Biophysics, University of Toronto, Toronto, Canada; Division of Physiology and Experimental Medicine, Hospital for Sick Children, Toronto, Canada

## Abstract

**Background:**

Phase contrast cardiovascular magnetic resonance (PC CMR) has emerged as a clinical tool for blood flow quantification but its use in the foetus has been hampered by the need for gating with the foetal heart beat. The previously described metric optimized gating (MOG) technique has been successfully used to measure foetal blood flow in late gestation foetuses on a 1.5 T CMR magnet. However, there is increasing interest in performing foetal cardiac imaging using 3.0 T CMR. We describe our pilot investigation of foetal blood flow measured using 3.0 T CMR.

**Methods:**

Foetal blood flows were quantified in 5 subjects at late gestational age (35–38 weeks). Three were normal pregnancies and two were pregnancies with ventricular size discrepancy. Data were obtained at 1.5 T and 3.0 T using a previously described PC CMR protocol. After reconstruction using MOG, blood flow was quantified independently by two observers. Intra- and inter-observer reproducibility of flow measurements at the two field strengths was assessed by Pearson correlation coefficient (R^2^), linear regression and Bland Altman analysis.

**Results:**

PC CMR flow measurements were obtained in 36 of 40 target vessels. Strong intra-observer agreement was obtained between measurements at each field strength (R^2^ = 0.78, slope = 0.83 ± 0.11), with a mean bias of −1 ml/min/kg and 95% confidence limits of ±71 ml/min/kg. Inter-observer agreement was similarly high for measurements at both 1.5 T (R^2^ = 0.86, slope = 0.95 ± 0.13, bias = 6 ± 52 ml/min/kg) and 3.0 T (R^2^ = 0.88, slope = 0.94 ± 0.13, bias = 4 ± 47 ml/min/kg). Across all PC CMR measurements, SNR per pixel was expectedly higher at 3.0 T relative to 1.5 T (165 ± 50%). The relative differences in flow measurements between observers were low (range: 4–16%) except for pulmonary blood flow which showed much higher variability at 1.5 T (34%) versus that at 3.0 T (11%). This was attributed to the poorly visualized, small pulmonary vessels at 1.5 T, which made delineation inconsistent between observers.

**Conclusions:**

This is the first pilot study to measure foetal blood flow using PC CMR at 3.0 T. The flow data obtained were in good correlation with those measured at 1.5 T, both within and between observers. With increased SNR at 3.0 T, smaller pulmonary vessels were better visualized which improved inter-observer agreement of associated flows.

**Electronic supplementary material:**

The online version of this article (doi:10.1186/s12968-015-0132-2) contains supplementary material, which is available to authorized users.

## Background

Ultrasound is an essential tool for the antenatal diagnosis of congenital heart disease, providing both anatomical and hemodynamic information at high temporal resolution. However, quantification of blood flow, a key hemodynamic parameter, is technically difficult by ultrasound. Inaccuracies arise from problems obtaining an appropriate angle of insonation, difficulty in measuring vessel area, and assumptions regarding the flow profile across the vessel lumen [1,2].

In recent years phase contrast cardiovascular magnetic resonance (PC CMR) has gained importance as a clinical tool for blood flow quantification in postnatal patients with cardiovascular disease [3] and does not have the same limitations as ultrasonography. Cardiovascular magnetic resonance (CMR) is a widely available and safe technique for imaging the foetus. However, it is hampered by the inability to accurately gate with the foetal heart beat resulting in inadequate spatial and temporal resolution. Our group has described a PC CMR technique with metric optimised gating (MOG) for use in late gestation foetal subjects [4,5], thus negating the need for ECG gating. This new technique has provided useful and novel information about blood flow in normal foetuses and those with left sided heart lesions [6]. However, the original technique and all hemodynamic flow data were obtained using a field strength of 1.5 T.

There is growing interest in utilizing 3.0 T systems for foetal imaging because signal to noise ratio (SNR) is improved, which can then be used to reduce scan time or obtain better spatial resolution. A recent qualitative comparison of foetal anatomical imaging at 1.5 T and 3.0 T supports this expectation [7]. Here we present a preliminary comparison of PC CMR blood flow measurement in the foetus at 1.5 T and 3.0 T, using MOG.

## Methods

All studies were approved by Toronto Hospital for Sick Children Research Ethics Board.

Foetal flow data were obtained from five subjects with gestational ages ranging from 35–38 weeks. The mothers were recruited following their 20 weeks anomaly ultrasound examination. In two cases, mild ventricular disproportion (right ventricle larger than left) was present, but resolved postnatally. The remaining three foetuses were structurally normal. None of these infants required surgery.

The CMR examinations were performed at 1.5 T and 3.0 T using Magnetom Avanto and Trio systems, respectively (Siemens Healthcare – Erlangen, Germany). The women were positioned in a lateral decubitus position for the scans, which was well tolerated. Data were acquired using surface coils (6 channel body matrix) in conjunction with elements from the spine array.

Four out of the five subjects were scanned on both scanners within the same day. For logistics reason, one patient had her second scan a week later. To avoid procedural bias, scanner order was alternated between subjects, and flow values were normalized by foetal body mass to account for differences in maturation.

Each CMR scan took up to a maximum of 60 minutes and was guided by the patient’s comfort level. Demographic data was collected and anonymized. The foetal heart rate was measured for 5 minutes using a cardiotocography (CTG) device (Corometrics, GE Healthcare – Fairfield, CT, USA) prior to CMR. The imaging protocol consisted of localizers in three orthogonal planes followed by a steady state free precession (SSFP) breath hold 3-dimensional acquisition of the whole foetus to estimate foetal weight, as described previously [8]. For prescription of the PC CMR scans, 3-plane static SSFP anatomical images were acquired through the foetal thorax (slice thickness 4 mm, slice gap 0.4 mm, repetition time 2.6 ms, echo time 1.14 ms, field of view 350 × 231 mm, matrix size 320 × 211 × 170, 1 signal average, Grappa acceleration factor 2). The PC CMR scan parameters were as follows: slice thickness 5 mm, echo time 2.92 ms, field of view 240 × 240 mm, matrix size 192 × 192, 33% phase oversampling, 1 signal average, no parallel imaging, 4 views per segment, temporal resolution 51.5 ms, 10 cardiac phases.

The following vessels were interrogated, equating to a maximum of 8 flow measurements per foetus: main pulmonary artery (MPA) (Additional files 1 and 2), branch pulmonary arteries (LPA & RPA) (Additional files 3 and 4), superior vena cava (SVC), arterial duct (AD), ascending aorta (AAo) (Additional files 5 and 6) and descending aorta at the diaphragm (DAo) using the anatomical images to plan the prescriptions as in post-natal PC CMR. The intra-abdominal portion of the umbilical vein (UV) proximal to the left portal branches was targeted to avoid complex flow behaviour. The velocity encoding range was tailored for the individual vessels with 150 cm/s for the MPA, AAo, AD, and DAo, 100 cm/s for the RPA, LPA and SVC and 50 cm/s for the UV.

Using analysis routines developed using a commercial data analysis package (MATLAB, MathWorks – Natick, MA, USA), the individual PC CMR measurements were reconstructed using MOG. The reconstructed PC CMR images were exported for flow quantification with regions of interest drawn around the vessels and flows measured by two independent observers using the commercially available cardiovascular post processing software Qflow (Medis – Leiden, NL). For body mass estimation, foetal volume was measured from the 3D SSFP data using Mimics (Materialise Group – Leuven, Belgium). Inter- and intra-observer reproducibility of flow measurements at the two field strengths was measured by Pearson correlation coefficient (R^2^), linear regression and Bland Altman analysis. Relative differences in flow between the two observers were also measured for each vessel (i.e., coefficient of variation). Finally, SNR per pixel was quantified for each vessel and at each field strength, defined as the vessel signal intensity (average over the ROI and through time) divided by the standard deviation of the signal intensity in uniform maternal fat near the receiver coil.

## Results

**Figure 1:**
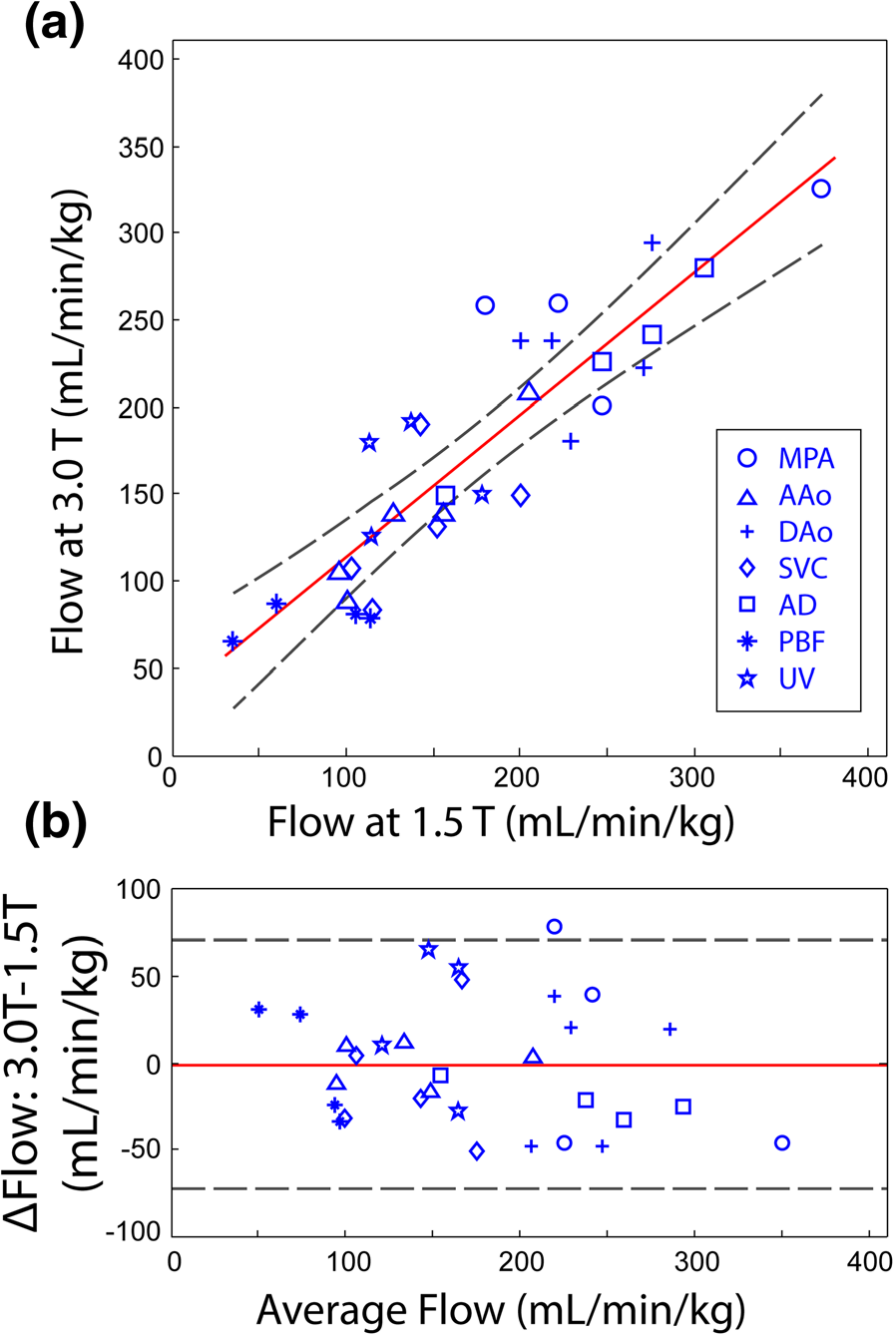
**Comparison of foetal flows measured at 3.0T versus 1.5T using PC CMR with MOG. (a)** Comparison of foetal flows measured at 3.0 T versus 1.5 T using PC CMR with metric optimized gating. Symbols = vessel type (see legend); Solid red line = linear regression; Dashed lines = 95% confidence limits **(b)** Bland-Altman analysis of data from **(a)**. Solid red line = mean; Dashed = 95% confidence limits.

PC CMR flow measurements were obtained in 36 of 40 target vessels. In one 3.0 T examination (structurally normal foetus), persistent foetal motion corrupted data from the AD, UV and pulmonary arteries. Figure 1a presents a correlation plot from the remaining flow comparisons, showing strong agreement between corresponding measurements at each field strength (R^2^ = 0.78, slope = 0.83 ± 0.16). Figure 1b is a Bland-Altman plot of the same data, with a mean bias of -1 ml/min/kg and 95% confidence limits of ±71 ml/min/kg. All measured flows are provided in Table 1.

**Table 1:** **Diagnosis, biometric data, and measured flows**

**Flow (mL/min/kg)**										
**Diagnosis**	**B0 (T)**	**GA (wk)**	**EFW (kg)**	**MPA**	**AAO**	**SVC**	**AD**	**DAO**	**PBF**	**UV**
RV/LV	1.5	36	3.20	181	96	153	248	230	106	179
Disproportion	3.0	35	2.87	259	106	133	227	182	82	151
RV/LV	1.5	38	3.25	373	101	143	306	271	60	116
Disproportion	3.0	38	3.24	327	89	191	281	223	88	126
Normal	1.5	37	3.74	218	157	104	191	219	68	119
	3.0	37	3.68	NR	140	108	NR	239	NR	NR
Normal	1.5	36	3.02	222	206	201	276	276	114	138
	3.0	36	2.80	261	209	150	243	295	80	192
Normal	1.5	38	3.55	248	128	116	158	201	35	115
	3.0	38	3.50	202	140	84	151	239	66	180

B0: field strength; GA: gestational age; EFW: estimated fetal weight; MPA: main pulmonary artery; AAO: ascending aorta; SVC: superior vena cava; AD: arterial duct; DAO: descending aorta; PBF: pulmonary blood flow; UV: umbilical vein; RV: right ventricle; LV: left ventricle; NR: Not Recorded due to foetal motion.

**Figure 2:**
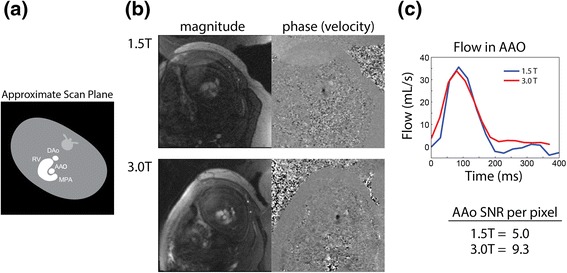
**Comparison of PC CMR of the AAo at 1.5 T and 3.0 T. (a)** Diagram of slice orientation through the foetal anatomy showing major vascular landmarks. **(b)** Magnitude and phase (velocity) data at 1.5 and 3.0 T, demonstrating superior SNR and anatomical visualization at 3.0 T. **(c)** Corresponding flow waveforms obtained from the AAo at 1.5 T (blue) and 3.0 T (red), and mean SNR (per pixel) for the AAo. RV = right ventricle.

**Figure 3:**
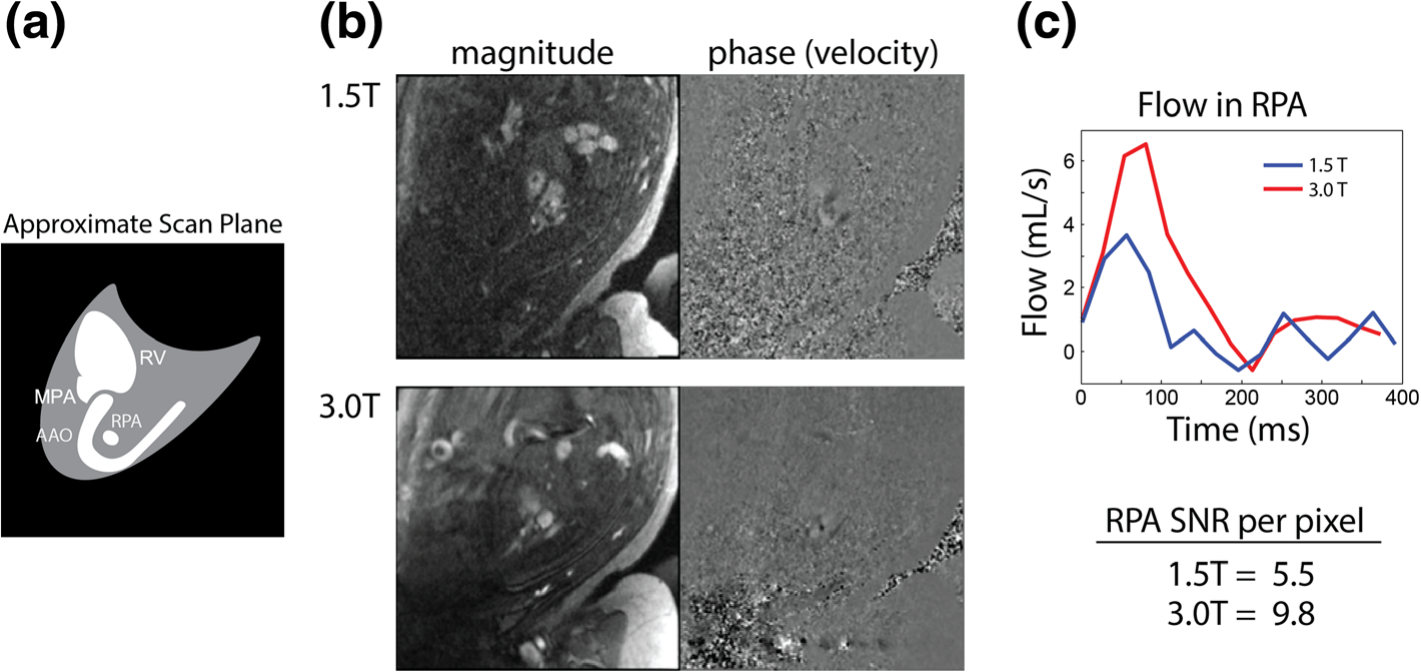
**Comparison of PC CMR of the RPA at 1.5 T and 3.0 T. (a)** Diagram of slice orientation through the foetal anatomy showing major vascular landmarks. **(b)** Magnitude and phase (velocity) data at 1.5 and 3.0 T, demonstrating superior SNR and anatomical visualization at 3.0 T. **(c)** Corresponding flow waveforms obtained from the RPA at 1.5 T (blue) and 3.0 T (red), and mean SNR (per pixel) for the RPA. RV = Right ventricle.

Inter-observer agreement was high for flows measured at 1.5 T (R^2^ = 0.86, slope = 0.95 ± 0.13, bias = 6 ± 52 ml/min/kg), with similar agreement at 3.0 T (R^2^ = 0.88, slope = 0.94 ± 0.13, bias = 4 ± 47 ml/min/kg). The relative difference in flow measurements between observers, by vessel and field strength, is provided in Table 2. Differences were generally low (4–16%), except for PBF which showed relatively high variability (34%) at 1.5 T versus that at 3.0 T (11%). This was attributed to the poorly defined, small pulmonary vessels at 1.5 T, which made consistent delineation difficult. To demonstrate this, representative PC CMR scans from the AAo and RPA of one subject are shown in Figures 2b and 3b, respectively. Qualitatively, better vessel depiction and higher SNR was provided by the 3.0 T scans. However, this difference in image quality did not translate to dramatic differences between the AAo flow waveforms (Figure 2c), whereas RPA flow was less noisy at 3.0 T. Across all measurements, SNR per pixel at 3.0 T increased by 165 ± 50% relative to 1.5 T.

**Table 2:** **Inter-observer flow coefficients of variation, by field strength and vessel type**

	**Coefficient of variation (%)**						
**B0 (T)**	**MPA**	**AAO**	**SVC**	**AD**	**DAO**	**PBF**	**UV**
1.5	8	5	16	13	4	34	7
3.0	4	10	11	9	10	11	14

B0: field strength; MPA: main pulmonary artery; AAO: ascending aorta; SVC: superior vena cava; AD: arterial duct; DAO: descending aorta; PBF: pulmonary blood flow; UV: umbilical vein.

## Discussion

The potential benefits of CMR as an adjunct to ultrasound for foetal assessment are well known, including improved tissue characterization, lack of a required acoustic window, and the opportunity to quantify blood flow and blood oxygen saturation [9]. However, the majority of foetal cardiovascular examinations have been performed using 1.5 T systems [10], including our own measurements of flow in foetuses with structurally normal hearts [5,11] and foetuses with left sided heart lesions [6]. In recent years, there has been increasing interest to perform foetal studies at higher field to shorten acquisition time or to improve spatial resolution. Victoria *et al.* recently published their experience imaging foetal structures using 1.5 T and 3.0 T systems [7]. Here, we investigated the benefits of higher field strength for dynamic foetal CMR, specifically foetal blood flow quantification, and observed SNR gains (165 ± 50%) in agreement with those reported for gradient-echo imaging in adults (160 – 170%) [12]. Because SNR also depended on many factors beyond field strength, such as maternal size and foetal position with respect to the receiver coils, average SNR gain was quoted rather than per vessel values.

This work provided an opportunity to quantify the reproducibility of foetal PC CMR between examinations, with results again comparable to studies previously performed in adults [13,14]. There was no significant difference in foetal mass values between the two systems. Similarly, measured flows were not significantly different between the two systems, and inter-observer agreement was high. However, flow waveforms in the smaller pulmonary vessels were more consistent between observers at 3.0 T, likely as a result of improved visualization of the smaller vessels provided by the increased SNR at higher field.

Although higher field strength improved SNR, it also posed its own challenges. First, we were mindful of the potential increase in acoustic noise levels associated with a 3.0 T system and the effect it may have on the mothers. Rapid switching of the magnetic field gradients is responsible for this acoustic noise, and the permissible sound level limit is currently set by the US Food and Drug Administration (FDA) over 140 dB [15]. We addressed this by providing appropriate ear plugs in addition to headphones, and subjects did not report any discernible difference in comfort level between the field strengths, although this issue was not specifically investigated. Second, energy deposition may be greater when scanning at higher field. Specific absorption rate (SAR) is a measure of energy deposition into a given mass during radiofrequency (RF) excitation. Numerical simulations have demonstrated that foetal SAR and temperature rise are within safety limits when scanning below < 2 W/kg whole body exposure [15,16]. In this study, no scans exceeded this recommended SAR. Finally, with increasing field strength there is an increased sensitivity to spatial signal variation due to susceptibility artifacts and standing wave phenomena. In this study, signal variation was most evident but tolerable at 3.0 T for SSFP imaging.

Limitations of this pilot study are as follows. First, changes in the physiological state of the foetus (and mother) between scans were an inevitable confounder. Second, in one foetus the scans were repeated one week apart rather than on the same day, which could further reduce overall agreement; however, this discrepancy was ameliorated by normalizing flow to foetal mass. Last, background phase correction was not applied to these interim results. Despite these limitations, flows obtained at 1.5 T and 3.0 T were in good agreement.

## Conclusion

Foetal flow measurements using PC CMR and MOG can be obtained at 1.5 T and 3.0 T with high correlation and negligible global bias. With increased SNR at 3.0 T, smaller pulmonary vessels were better visualized which improved inter-observer agreement of associated flows. This feasibility study is encouraging given the growing interest in 3.0 T CMR for foetal anatomical and functional cardiac imaging.

## Additional files

Additional file 1:
**PC CMR of the fetal main pulmonary artery (MPA) at 1.5T.** Video showing magnitude and phase (velocity) data acquired from the fetal MPA using PC CMR at 1.5T. Additional file 2:
**PC CMR of the fetal main pulmonary artery (MPA) at 3.0T.** Video showing magnitude and phase (velocity) data acquired from the fetal MPA using PC CMR at 3.0T. Additional file 3:
**PC CMR of the fetal right pulmonary artery (RPA) at 1.5T.** Video showing magnitude and phase (velocity) data acquired from the fetal RPA using PC CMR at 1.5T. Additional file 4:
**PC CMR of the fetal right pulmonary artery (RPA) at 3.0T.** Video showing magnitude and phase (velocity) data acquired from the fetal RPA using PC CMR at 3.0T. Additional file 5:
**PC CMR of the fetal ascending aorta at 1.5T.** Video showing magnitude and phase (velocity) data acquired from the fetal ascending aorta (AAO) using PC CMR at 1.5T. Additional file 6:
**PC CMR of the fetal ascending aorta at 3.0T.** Video showing magnitude and phase (velocity) data acquired from the fetal ascending aorta (AAO) using PC CMR at 3.0T.
